# Promoting Awareness of Data Confidentiality and Security During the COVID-19 Pandemic in a Low-Income Country—Sierra Leone

**DOI:** 10.3389/phrs.2024.1607540

**Published:** 2024-11-08

**Authors:** Joseph Sam Kanu, Mohamed A. Vandi, Brima Bangura, Katherine Draper, Yelena Gorina, Monique A. Foster, Jadnah D. Harding, Eric N. Ikoona, Amara Jambai, Mohamed A. M. Kamara, Daniel Kaitibi, Daphne B. Moffett, Tushar Singh, John T. Redd

**Affiliations:** ^1^ Directorate of Health Security and Emergencies, Ministry of Health, Freetown, Sierra Leone; ^2^ Infectious Diseases and Outbreaks Division, Alabama Department of Public Health, Montgomery, AL, United States; ^3^ Global Health Center, Centers for Disease Control and Prevention, Atlanta, GA, United States; ^4^ Department of Physics and Computer Science, School of Technology, Njala University, Freetown, Sierra Leone; ^5^ The Schmidt Initiative for Long Covid (SILC), Menlo Park, CA, United States

**Keywords:** data confidentiality, data security, data ownership, cybersecurity, training, Sierra Leone, Sierra Leone Epidemiological Data Team, SLED

## Abstract

**Objectives:**

World Health Organization issued Joint Statement on Data Protection and Privacy in the COVID-19 Response stating that collection of vast amounts of personal data may potentially lead to the infringement of fundamental human rights and freedoms. The Organization for Economic Cooperation and Development called on national governments to adhere to the international principles for data security and confidentiality. This paper describes the methods used to assist the Ministry of Health in bringing awareness of the data ownership, confidentiality and security principles to COVID-19 responders.

**Methods:**

The Sierra Leone Epidemiological Data (SLED) Team data managers conducted training for groups of COVID-19 responders. Training included presentations on data confidentiality, information disclosure, physical and electronic data security, and cyber-security; and interactive discussion of real-life scenarios. A game of Jeopardy was created to test the participant’s knowledge.

**Results:**

This paper describes the methods used by the SLED Team to bring awareness of the DOCS principles to more than 2,500 COVID-19 responders.

**Conclusion:**

Similar efforts may benefit other countries where the knowledge, resources, and governing rules for protection of personal data are limited.

## Introduction

In November 2020, the World Health Organization issued Joint Statement by United Nations System Organizations on Data Protection and Privacy in the COVID-19 Response stating that collection of vast amounts of personal and sensitive data may potentially lead to the infringement of fundamental human rights and freedoms [1]. The Organization for Economic Cooperation and Development called on national governments to adhere to the international principles for data security and confidentiality [2, 3].

Sierra Leone is a West African country of 7.5 million people and is divided into sixteen districts and five regions [4]. Sierra Leone ranks 184 out of 193 countries and territories on the 2022 Human Development Index and is classified as low-income country by The World Bank [5, 6]. The healthcare system is resource-constrained and challenged by frequent infectious disease outbreaks, the 2014–2016 epidemic of Ebola Virus Disease, and the COVID-19 pandemic [7]. The Ministry of Health (MoH) oversees 29 hospitals and more than 1,200 peripheral health units [8].

The first case of COVID-19 was reported on the 30th of March 2020. It was followed by the national epidemic response measures, including lockdown, travel bans, and curfews [9, 10]. To promote data ownership, confidentiality, and security (DOCS) awareness, the MoH initiated training on the DOCS principles and issued a Data Security and Confidentiality Agreement for MoH COVID-19 responders. The training sessions were conducted by the Sierra Leone Epidemiological Data (SLED) Team. Since 2017, the SLED Team of Sierra Leonean data managers that was created by the MoH and supported by the US Centers for Disease Control and Prevention (CDC), has been assisting with management and use of the Sierra Leone Ebola Database (Database) and helping families through a family reunification program to identify graves of their loved ones who were lost during the epidemic [11, 12]. Since its inception, the SLED Team was trained on identification of personally identifiable information (PII) and DOCS by MoH and CDC specialists and through online courses, which enabled them to develop training materials to establish and strengthen this expertise more broadly in the country.

This paper describes the methods used by the SLED Team to assist the MoH in bringing awareness of the DOCS principles to MoH COVID-19 responders. Similar efforts may benefit other countries where the knowledge, resources, and governing rules for protection of personal data are limited [13].

## Methods

### Training Materials

The DOCS materials were developed based on the training provided as part of 2018 3-day workshop for the Sierra Leoneans. DOCS sessions included presentations on PII recognition, principles of confidentiality and data ownership, information disclosure, electronic and physical data security, with discussions of real-life scenarios. Later, the DOCS sessions were delivered as a part of data management, surveillance, emergency management and other trainings to COVID-19 responders lasting 30 min to 3 h. A training module on principles of cyber security and an optional DOCS Jeopardy game were added to the training. To deliver basic DOCS information, the duration of a training session needs to be 2 h with 30 min for discussion.

### DOCS Jeopardy

A game of DOCS Jeopardy was created to test the participant’s knowledge and encouraged the attendees to think deeply about real-life DOCS situations. The game included the following four categories: Confidentiality, Disclosure, Physical and Electronic Security, and PII. Generally, each question was based on a real-life scenario, such as, “You are a medical doctor in Connaught Hospital and one of your HIV positive patients proposed to your sister but has never revealed this information to her. What should you do?” Attendees participated in Jeopardy as individuals, but also worked in teams to answer the questions. The participants were awarded symbolic prizes, usually a piece of candy, for correct answers to encourage engagement. The DOCS Jeopardy game was received with great enthusiasm and resulted in thoughtful discussions. The DOCS Jeopardy game takes 1–3 h and was not always conducted due to allotted time constraints.

### Data Security and Confidentiality Agreement

To ensure that COVID-19 responders understand their responsibility for unauthorized disclosure of PII, the MoH established a Data Security and Confidentiality Agreement that is required to be signed by attendees of DOCS training sessions. The Agreement states basic requirements for confidentiality protection, such as “I will not make any unauthorized copy, transfer, modification or purging of confidential information.” It concludes with a note about disciplinary measures that may be taken if these requirements are willfully violated. Signed by the attendee, the agreements were collected by the supervisors. The text of the Data Security and Confidentiality Agreement is in Supplementary Material S1.

### Memory Card

A Questions-and-Answers (Q&A) Memory Card was created, containing answers to typical questions asked by participants during the training sessions, such as “Why is the training on data ownership, confidentiality, and security being offered now?” The Q&A Memory Card was distributed at the end of each session for use by the attendees and for sharing with their co-workers. The Q&A Memory Card is presented in the Supplementary Material S2.

### Training Evaluation Form

To assess pre-training knowledge, needs for training improvement and to understand challenges in maintaining DOCS principles, at the beginning and at the end of each session the SLED Team distributed a Training Evaluation Forms (Form). Completing the Form was voluntary and anonymous. The Form contains questions such as whether the presenter was engaging and easy to follow, or if the attendee would be able to maintain DOCS principles, and, if not, what were their challenges in maintaining DOCS. A template of the Training Evaluation Form is in Supplementary Material S3.

### Training Report

After each DOCS session, one of the team members developed a training report that included date, place, and duration of the training, name of the organization, description of the attendees’ occupations, the questions asked and answers provided, and participants feedback and comments. These reports were used to identify gaps in knowledge, the need for data protection infrastructure, and to improve training delivery. A template of the Training report is in Supplementary Material S4.

### Tracking System

A tracking system was created to keep records of completed DOCS training sessions and for planning purposes. The tracking system captures the following data elements: contact person name and email, requesting organization, address, and time of the training, planned and actual number of attendees, attendees’ occupation, and comments.

### Training Under COVID-19 Circumstances

Each training was conducted following COVID-19 precautions, such as social distancing and wearing masks. Online training to the attendees in the distant districts was conducted at the early stage of epidemic.

## Results

By October 2023, the SLED Team trained more than 2,500 healthcare workers and COVID-19 responders. The DOCS training was conducted as a part of the MoH, CDC, WHO, UNICEF, African Field Epidemiology Network (AFENET), and other organizations’ trainings or workshops. The number of participants varied from 7 to 110, and included district medical officers, surveillance officers, data managers, laboratory sample collectors, and other healthcare workers and COVID-19 responders. All filled out pre-training evaluation forms (n = 468) indicated low or no knowledge of the DOCS principles with comments such as “No knowledge in data security and confidentiality” or “I have been uncertain about how to securely share sensitive data with colleagues.”

In 33% (372 out of 1,110) of post-training evaluation forms, participants noted that they would like to have a follow-up training and gain more knowledge of DOCS. At each session, attendees expressed interest in the topic and initiated discussions about their ability and challenges to maintain the DOCS principles. These discussions led to development of the Q&A Memory Card and the Evaluation Form with a goal to assess major challenges.

Multiple concerns were expressed by the participants. Data ownership was a difficult concept for participants to understand, some believed that everyone working with data should be a data owner. Many participants described their challenges with electronic data security, from difficulties with memorizing changing passwords to the lack of secure file transfer protocols or encryption for safe data transfer. Physical security was also a challenge in some settings, such as no locked cabinets for the hard copies. Some participants were concerned that their supervisors may not be aware of the DOCS principles and may not appreciate their data security protection efforts.

Although no evaluation of the training impact on improving data confidentiality was conducted, many participants offered suggestions on improving and expanding the scope of the DOCS training program, such as refresher training sessions, national DOCS educational program for all healthcare workers, appointment of MoH data confidentiality officers, and inclusion of DOCS in educational curriculum.

Online training that initially was provided to attendees in the distant districts experienced frequent interruption due to poor internet connection and other technical issues. These factors, and the novelty of the software and of distant learning for most of the participants, resulted in the loss of motivation and cancellation of online session. In these cases, instructors who were present in the training room switched to another training topic. As a result, online training was discontinued and only in-person training was provided.

## Discussion

This work documents and underscores the need to promote awareness of data confidentiality and security throughout the COVID-19 pandemic in Sierra Leone and provides details of trainings on the principles of ownership of data, maintaining data confidentiality and security. Starting with the early days of the COVID-19 pandemic, the SLED Team was able to train over 2,500 COVID-19 responders. Participants appreciated the trainings as they had low knowledge on DOCS prior to the trainings. They were enthusiastic in their motivation to apply the principles of DOCS and recommended dissemination of the trainings to all healthcare workers in the country.

Maintaining PII confidentiality is mandated by the Constitution of Sierra Leone [14], however, as in many other African countries [13], the rules governing the protection of personal data and mechanisms for enforcing them are limited. In this digital age, vast amounts of patient data are captured electronically. Although electronic data has its advantages, it also poses the challenge of maintaining its confidentiality. This challenge is more pronounced in responding to healthcare emergencies, such as Ebola Virus Disease or COVID-19. Previous studies [15, 16] have extensively described patients’ concerns about unauthorized information disclosure. Having knowledge of these concerns and the DOCS principles is critical for healthcare workers.

The DOCS training program presents a solution to build an awareness of the responsible use of personal data to serve the essential need for data sharing. More efforts should be directed to implementation of these principles in the healthcare data managing practice.

## References

[1] World Health Organization. Joint Statement on Data Protection and Privacy in the COVID-19 Response (2020). Available from: https://www.who.int/news/item/19-11-2020-joint-statement-on-data-protection-and-privacy-in-the-covid-19-response (Accessed May 2, 2023). PMC8015502 33530055

[2] Organization for Economic Co-operation and Development. Ensuring Data Privacy as We Battle COVID-19 (2020). Available from: https://www.oecd-ilibrary.org/industry-and-services/ensuring-data-privacy-as-we-battle-covid-19_36c2f31e-en (Accessed October 28, 2024).

[3] World Health Organization. World Health Organization Data Principles (2020). Available from: https://www.who.int/data/principles (Accessed May 2, 2023).

[4] Statistics Sierra Leone. Sierra Leone Demographic and Health Survey 2019 (2020). Available from: https://www.statistics.sl (Accessed December 15, 2023). 10.1111/j.1728-4465.2011.00265.x 21500702

[5] United Nations Development Programme (UNDP). Human Development Report 2023/2024 (2024). Available from: https://hdr.undp.org/system/files/documents/global-report-document/hdr2023-24reporten.pdf (Accessed October 11, 2024).

[6] World Bank. The World by Income and Region. The World Bank Group (2024). Available from: https://datatopics.worldbank.org/world-development-indicators/the-world-by-income-and-region.html (Accessed October 10, 2024).

[7] UNICEF. Emergency Preparedness and Response in Sierra Leone (2024). Available from: https://www.unicef.org/sierraleone/emergencies (Accessed September 23, 2023).

[8] Government of Sierra Leone. Ministry of Health Website (2024). Available from: https://mohs.gov.sl/ (Accessed October 10, 2023).

[9] Stone H Bailey E Wurie H Leather AJM Davies JI Bolkan HA A Qualitative Study Examining the Health System’s Response to COVID-19 in Sierra Leone. PLoS ONE (2024) 19(2):e0294391. 10.1371/journal.pone.0294391 38306321 PMC10836672

[10] Parmley LE Hartsough K Eleeza O Bertin A Sesay B Njenga A COVID-19 Preparedness at Health Facilities and Community Service Points Serving People Living with HIV in Sierra Leone. PLoS ONE (2021) 16(4):e0250236. 10.1371/journal.pone.0250236 33857253 PMC8049332

[11] Agnihotri S Alpren C Bangura B Bennett S Gorina Y Harding JD Building the Sierra Leone Ebola Database: Organization and Characteristics of Data Systematically Collected during 2014-2015 Ebola Epidemic. Ann Epidemiol (2021) 60:35–44. 10.1016/j.annepidem.2021.04.017 33965545

[12] Bensyl D Bangura B Cundy S Gegbai F Gorina Y Harding JD Finding the Graves: SLED Family Reunification Program. Ann Epidemiol (2021) 64:15–22. 10.1016/j.annepidem.2021.05.003 34058352 PMC10860640

[13] Daigle B. Data Protection Laws in Africa: A PanAfrican Survey and Noted Trend. J Int Commerce Econ (2021) 2:1–27.

[14] Government of Sierra Leone. The Sierra Leone Constitution 1991 (1991). Available from: http://www.sierra-leone.org/Laws/constitution1991.pdf (Accessed April 17, 2023).

[15] Moulaei K Iranmanesh E Amiri P Ahmadian L. Attitudes of COVID‐19 Patients toward Sharing Their Health Data: A Survey‐based Study to Understand Security and Privacy Concerns. Health Sci Rep (2023) 6:e1132. 10.1002/hsr2.1132 36865528 PMC9971706

[16] Trestian R Xie G Lohar P Celeste E Bendechaet M Brennan R Privacy in a Time of COVID-19: How Concerned Are You? IEEE Security and Privacy (2021) 19(5):26–35. 10.1109/msec.2021.3092607

