# Supplementary material for: Promoting Awareness of Data Confidentiality and Security During the COVID-19 Pandemic in a Low-Income Country—Sierra Leone
Source: Public Health Rev. 2024 Nov 8;45:1607540. doi: 10.3389/phrs.2024.1607540 (PMC11581828; doi:10.3389/phrs.2024.1607540)
Supplement: Supplementary file 1 [file DataSheet2.pdf]

# Memory Card

# Data Ownership, Confidentiality and Security (DOCS) and Cybersecurity

**Background.** Confidentiality is the assurance or promise not to release **Personally Identifiable Information (PII)** in any way that will allow the person or establishment to be identified. PII refers to data that can be used to identify, locate, or contact individuals, or reveal the characteristics or other details about them. It might consist of **direct identifiers**, such as the name, address or other information that is unique to an individual, or **indirect identifiers** such as extreme age, unusual occupation district or chiefdom, or information from other sources such as work roster. Unauthorized disclosure of PII and private information and ineffectiveness to protect the confidentiality of patient's health information may result in the termination of employment or legal actions.

**Question.** What is data security?
**Answer.** Data security refers to the protection of data information from unauthorized access, use, disclosure, alteration, or destruction. It involves implementing measures to ensure the confidentiality, integrity, and availability of data.

**Question.** Why is data security important?
**Answer.** Data security is crucial because it helps protect sensitive and valuable information from unauthorized access or misuse. It helps maintain the privacy of personal data, prevents data breaches, safeguards intellectual property, and preserves the trust of patients.

**Question.** What are some common threats to data security?
**Answer.** Common threats to data security include negligence to protect the data, limited options to secure the data, data theft, theft or loss of devices, insecure network connections, phishing scams, and hacking attacks.

**Question.** Is it right for a data manager/data collector to share confidential data with other staff in the same organization?
**Answer.** Data manager or data collector should not share the data unless that staff is authorized to have access to the data. Data sharing should be done solely on an "as needed" basis.

**Question.** What are some best practices for data security?
**Answer.** Some best practices for data security include training employees on security awareness, establishing data security policies and appointing confidentiality officers, keeping paper forms with PII in secure file cabinets, implementing strong passwords for electronic devices, encrypting PII electronic data, using firewalls and antivirus software, and monitoring and logging system activities. Organizations should establish procedures for reporting and managing a suspected data security breach.

**Question.** Why do we need to protect the data when sometimes the confidential information is well known by the community? For example, if someone got sick in the village why do we need to protect this information?
**Answer.** We must protect PII data because this information should not be shared with other people or organizations that are not a part of the community. PII data must be coded before sharing. Only those who are authorized and need to know the data for their duties should have access to them. Everyone who has access to the data should justify a reason for the data use.

**Question.** Is it wrong for a data owner to sell his/her data to make money?
**Answer.** The organization that is the data owner should make this decision BEFORE the data are collected and it should be specified in all agreements and documents. The persons that supply PII data should agree that their information will be sold. Unless an arrangement is made beforehand and a person gives his/her permission, it is wrong and may lead to termination of employment and legal actions.

**Question.** Is it right to take a snapshot of a patient in a facility and publicize it on social media with his information and medical conditions?
**Answer.** No, it is not right because it was not with the patient's consent and its unauthorized disclosure, intentional to gain something. This action may result in the termination of employment or legal actions.

**Question.** Can surveillance data be categorized as classified data set?
**Answer.** Yes, surveillance data must be categorized as classified data sets if they contain PII information.

**Question.** What is the best way to share the PII data? Is it right to transfer PII data through email?
**Answer.** A secure file transfer protocol (sFTP) system or any other secure technology is the best way to share PII data. Un-encrypted email is not a secure way to share data. Never send the data by email with the password attached.

**Question.** What are the best ways to secure PII data in the office?
**Answer.** Hard (paper) copies are best secured in locked cabinets with access available only to authorised persons. Soft (electronic) copies must be protected using physical and electronic security tools, such as locking computers, up-to-date antivirus software, and other measures that you learned from the training.

**Question.** Who is responsible for data storage, safety, and distribution?
**Answer.** Everyone who is eligible to work with the PII data is responsible for data security. Data Custodian is responsible for data storage, safety, and distribution and may have physical possession of the data. Data Owner makes decisions about all issues related to data distribution and resolution of the data breaches.

**Question.** Can someone access PII data for their work without the approval of the data owner or data custodian?

**Answer.** Nobody should access PII unless they are authorized, and they or their organization has an agreement with the Data Owner. Data User Agreement must contain conditions and details of access. Access to PII data should be kept to a minimum.

**Question.** Are there data protection regulations in Sierra Leone?
**Answer.** The Constitution's Article 25(2)(a)(ii), under the protection of freedom of expression and the press, prevents the "disclosure of information received in confidence." The Right to Access Information Law under Article 21 restricts the disclosure of information if it relates to personal information concerning an individual, with certain exemptions relating to the prior consent of the individual or if the information is necessary for public health, safety, among others (21(2)).

**Question.** What is encryption, and how does it enhance data security?
**Answer.** Encryption is the process of converting data into a secure form using algorithms to make it unreadable to unauthorized individuals. It enhances data security by ensuring that even if data are intercepted or stolen, they remain protected and cannot be accessed without the encryption key.

**Question.** What steps should be taken in the event of a data breach?
**Answer.** In the event of a data breach, steps should include:

1. An immediate report to the supervisor,
2. Conducting a thorough investigation,
3. Notifying the appropriate authorities and affected individuals,
4. Implementing measures to prevent similar incidents in the future.
5. In the event of an electronic data breach, in addition, an immediate report to the IT personnel and isolating affected systems is needed. Organizations should develop procedures for reporting and managing a suspected data security breach.

**Question.** What should individuals do to protect their personal data?
**Answer.** Individuals can protect their personal data by keeping physical PII data in a secure place, using strong and unique passwords for electronic devices, being cautious about sharing personal information online, regularly updating software and devices, being vigilant against phishing attempts, using secure Wi-Fi networks, and regularly reviewing privacy settings on online accounts.

**Question:** What is the primary goal of physical security?
**Answer:** The primary goal of physical security is to prevent unauthorized access to the documents, protect assets, and ensure the safety of people within a given physical space.

**Question**: What challenges can impact the effectiveness of physical security?
**Answer:** Several challenges can affect physical security, including:

- Human Error: Employees failing to follow security procedures or accidentally leaving doors unlocked.
- Insider Threats: Malicious actions by individuals with authorized access.
- External Threats: Criminal activities, natural disasters, or terrorist acts that can bypass security measures.

**Question:** Why is physical security important for healthcare personnel and facilities?
**Answer:** Physical security is crucial for healthcare facilities for several reasons:

- Personnel Safety: It ensures the safety of employees, visitors, and patients by preventing unauthorized individuals from entering sensitive areas.
- Data Protection: Physical security measures help prevent physical access to servers and other storage devices, and paper forms that contain sensitive data, reducing the risk of data breaches.

**Question**: What is cybersecurity in the context of healthcare?
**Answer**: Cybersecurity in the context of healthcare refers to the protection of sensitive patient information, medical records, and other confidential data from unauthorized access, theft, or damage in cyberspace. It involves a set of technologies, practices, and policies designed to secure computer networks, devices, and data against cyber threats such as malware, ransomware, phishing attacks, and hacking.

**Question**: Why is cybersecurity important in healthcare?
**Answer**: Cybersecurity is important in healthcare because patient data are highly sensitive and valuable, and breaches can lead to serious consequences, including identity theft, financial fraud, reputational damage, and even physical harm to the patient. In addition, healthcare organizations are increasingly reliant on digital technologies to deliver care, making them more vulnerable to cyber threats.

**Question**: What are some common cyber threats to healthcare organizations?
**Answer**: Some common cyber threats to healthcare organizations include phishing attacks, ransomware, malware, distributed denial-of-service (DDoS) attacks, and insider threats. These threats can be initiated by external factors such as hackers, or by internal actors such as employees or contractors with malicious intent.

**Question**: How can healthcare organizations prepare for cyber-attacks?
**Answer**: Healthcare organizations can prepare for cyber-attacks by:

1. Developing and implementing a comprehensive cybersecurity strategy that includes risk assessments, incident response plans, and regular training for employees.
2. Establishing a culture of cybersecurity awareness and accountability across the organization.
3. Developing and maintaining a robust backup and disaster recovery system to ensure that critical data and systems can be restored quickly in the event of an attack.
4. Partnering with trusted cybersecurity vendors and consultants to ensure that the organization has access to the latest threat intelligence and best practices.

**Question.** Can an organization request training on data ownership, confidentiality, and security?
**Answer.** Yes, the organization interested in training on data ownership, confidentiality, and security should contact the SLED Data Confidentiality Officer.
