# Supplementary material for: Promoting Awareness of Data Confidentiality and Security During the COVID-19 Pandemic in a Low-Income Country—Sierra Leone
Source: Public Health Rev. 2024 Nov 8;45:1607540. doi: 10.3389/phrs.2024.1607540 (PMC11581828; doi:10.3389/phrs.2024.1607540)
Supplement: Supplementary file 2 [file DataSheet4.PDF]

[Title of the training]

[Place of the training]

Introduction:

[Brief description of the training]

Training objectives:

Organizer: [Organization that requested and funded the training]

When: [Dates of the training]

Where: [District, town, venue of the training]

Training facilitators:

<u>Session 1</u>

Session date:

Session time and duration:

Topic(s) presented:

Attendees: [General description of the participants]

Number of attendees:

Specialties of attendees:

Presenter/facilitator:

[Note: repeat if multiple sessions were presented.

SLED presentation on Data Ownership, Confidentiality, and Security (DOCS):
[Brief description]

Attendees' participation:
[Facilitator' assessment of how participants reacted]

Attendees' feedback:

- [Document oral comments by the participants]

Questions and Answers:

- [Document questions asked by the participants and answers given]

Photos (if taken):
