# Supplementary material for: Promoting Awareness of Data Confidentiality and Security During the COVID-19 Pandemic in a Low-Income Country—Sierra Leone
Source: Public Health Rev. 2024 Nov 8;45:1607540. doi: 10.3389/phrs.2024.1607540 (PMC11581828; doi:10.3389/phrs.2024.1607540)
Supplement: Supplementary file 3 [file DataSheet3.PDF]

Training Evaluation Form

Title of event: Data Ownership, Confidentiality, and Security Awareness (DOCS)

Date of event: <Date>

Location of event: <Location>

Trainers: <SLED Team member(s)>

**Instructions:** Please choose your level of agreement	Agree	Disagree	COMMENTS
1. Principles of data confidentiality and security are clear			
2. The presenters were engaging			
3. The content was organised and easy to follow			
4. The trainers were well prepared and able to answer questions			
5. The course length was appropriate			

6. What was most useful/applicable for your work?

7. Would you be able to maintain data confidentiality and security in your work? If not, please explain in the box below.

Yes
No

7a. I would not be able to maintain data confidentiality and security in my work because… (please write below)

8. Please provide any other comments or suggestions about the Data Ownership, Confidentiality, and Security

THANK YOU FOR COMPLETING THIS EVALUATION FORM. FEEDBACK RECEIVED WILL BE USED TO PROVIDE IMPROVEMENTS TO FUTURE EVENTS.

PLEASE HAND THE FILLED FORM TO THE TRAINERS.

ADDITIONAL COMMENTS OR QUESTIONS CAN BE SEND TO THE SLED TEAM VIA EMAIL
