# Supplementary material for: Promoting Awareness of Data Confidentiality and Security During the COVID-19 Pandemic in a Low-Income Country—Sierra Leone
Source: Public Health Rev. 2024 Nov 8;45:1607540. doi: 10.3389/phrs.2024.1607540 (PMC11581828; doi:10.3389/phrs.2024.1607540)
Supplement: Supplementary file 4 [file DataSheet1.PDF]

Government of Sierra Leone

Ministry Of Health and Sanitation

Data Security and Confidentiality Agreement

(to be signed after attending training session on data confidentiality and security)

Name (print):____ Date: _____________

Title/Position:____

Employer: ____

Department: ____

Confidentiality is the assurance or promise not to release **Personally Identifiable Information (PII)** in any way that will allow the person or establishment to be identified. PII refers to data that can be used to identify, locate, or contact individuals or establishments, or reveal the characteristics or other details about them. It might consist of **direct identifiers**, such as the name, address or other information that is unique to an individual, or **indirect identifiers** such as extreme age, unusual occupation, or information from other sources such as work roster etc.
All documents and messages created, sent, or retrieved using any communication or computer system is the property of the organisation I am working for. Unauthorised by my employer disclosure of PII and private information and ineffectiveness to protect the confidentiality of their health information may result in the termination of my employment.
During my employment/assignment, I understand that I may come into the possession of Confidential Information related to patients. I understand that patient information is private, whereby the confidentiality is protected by the Constitution of Sierra Leone.

I will maintain the confidentiality of all data and information to which I have access, whether that access was provided on site or remotely. I further understand that I must sign and comply with this agreement to get authorization for access to any Confidential Information. By signing this document, I understand and hereby agree to the following:

**Item**	**Check**
1. I will comply with all security and privacy policies to protect the security and privacy of Confidential Information.	☐
2. I will not access or view any Confidential Information, other than what is required to do my job.	☐

3. I will not email, save or transfer Confidential Information unless it is specifically authorized by my employer and consistent with policies and procedures to protect and secure it. ☐
4. I will not disclose or discuss any Confidential Information with others, including friends and family, who do not have a “business need to know”. ☐
5. I will not discuss Confidential Information when others can overhear the conversation (for example, in hallways, or on public transportation) It is not acceptable to discuss Confidential Information in public areas even if a patient’s name is not used. ☐
6. I will not send any fraudulent, harassing, or obscene messages or attachments to messages through any communication system. ☐
7. I will not make any unauthorized copy, transfer, modification or purging of Confidential Information. ☐
8. I will log off any computer prior to leaving it unattended and follow proper shut-down procedures at the end of my shift. ☐
9. I will not disclose my computer ID and password to another party or knowingly use another person’s computer ID and password instead of my own for any reason. In addition, I understand that my personal user ID(s), and passwords used to access computer systems are also an integral aspect of the Confidential Information. ☐
10. Upon termination of my employment, I will immediately return any documents or other media containing Confidential Information ☐
11. will immediately report to my supervisor any activity, by any person, including myself, that is a violation of this Agreement or breach of confidentiality. ☐

I understand that violation of this Agreement may result in disciplinary action, up to and including termination of employment or contract and/or suspension and loss of privileges, as well as legal liability.

I have read the above agreement and agree to comply with all its terms.

Signature:____Date:____________________

Witness:____Date:____________________
